# Impact of Aggregation Methods for Texture Features on Their Robustness Performance: Application to Nasopharyngeal ^18^F-FDG PET/CT

**DOI:** 10.3390/cancers15030932

**Published:** 2023-02-01

**Authors:** Lihong Peng, Hui Xu, Wenbing Lv, Lijun Lu, Wufan Chen

**Affiliations:** 1 School of Biomedical Engineering, Southern Medical University, Guangzhou 510515, China; 2 Guangdong Provincial Key Laboratory of Medical Image Processing, Southern Medical University, Guangzhou 510515, China; 3 Guangdong Province Engineering Laboratory for Medical Imaging and Diagnostic Technology, Southern Medical University, Guangzhou 510515, China; 4 Department of Electronic Engineering, Information School, Yunnan University, Kunming 650504, China; 5 Pazhou Laboratory, Guangzhou 510330, China

**Keywords:** radiomics, PET/CT, robustness, texture feature

## Abstract

**Simple Summary:**

This study investigates the impact of aggregation methods used for the generation of texture features on their robustness of nasopharyngeal carcinoma (NPC) based on ^18^F-FDG PET/CT images. 128 NPC patients were enrolled and 95 texture features were extracted for each patient including six feature families under different aggregation methods. For GLCM and GLRLM features, six aggregation methods were considered. For GLSZM, GLDZM, NGTDM and NGLDM features, three aggregation methods were considered. The robustness of the feature was assessed by the intra-class correlation coefficient (ICC). Different dimensional features with same aggregation methods showed worse robustness compared with the same dimensional features with different aggregation methods. Different discretization levels and PVC algorithms have a negligible effect on the percent of ICC categories of all texture features.

**Abstract:**

Purpose: This study aims to investigate the impact of aggregation methods used for the generation of texture features on their robustness of nasopharyngeal carcinoma (NPC) based on ^18^F-FDG PET/CT images. Methods: 128 NPC patients were enrolled and 95 texture features were extracted for each patient including six feature families under different aggregation methods. For GLCM and GLRLM features, six aggregation methods were considered. For GLSZM, GLDZM, NGTDM and NGLDM features, three aggregation methods were considered. The robustness of the features affected by aggregation methods was assessed by the pair-wise intra-class correlation coefficient (ICC). Furthermore, the effects of discretization and partial volume correction (PVC) on the percent of ICC categories of all texture features were evaluated by overall ICC instead of the pair-wise ICC. Results: There were 12 features with excellent pair-wise ICCs varying aggregation methods, namely joint average, sum average, autocorrelation, long run emphasis, high grey level run emphasis, short run high grey level emphasis, long run high grey level emphasis, run length variance, SZM high grey level emphasis, DZM high grey level emphasis, high grey level count emphasis and dependence count percentage. For GLCM and GLRLM features, 19/25 and 14/16 features showed excellent pair-wise ICCs varying aggregation methods (averaged and merged) on the same dimensional features (2D, 2.5D or 3D). Different discretization levels and partial volume corrections lead to consistent robustness of textural features affected by aggregation methods. Conclusion: Different dimensional features with the same aggregation methods showed worse robustness compared with the same dimensional features with different aggregation methods. Different discretization levels and PVC algorithms had a negligible effect on the percent of ICC categories of all texture features.

## 1. Introduction

Positron emission tomography (PET) has been established as a powerful quantitative imaging technique [1]. For PET imaging, standardized uptake value (SUV) based parameters are the most commonly adopted image biomarkers (such as SUVmean and SUVmax) in routine clinical application for diagnostic and prognostic assessment [2]. Due to good reproducibility or robustness, SUV parameters have been extensively used in multicenter studies [1,3], but these parameters are limited by the insufficient descriptions of tumor heterogeneity [4]. With the development of radiomics, the image biomarkers are extended beyond the SUV to a large set of radiomic features, which are achieved by using statistical, shape-based, and/or textural features, including second- and higher-order methods of increasing complexity [5,6,7,8]. The reproducibility and robustness of radiomic features are very critical to radiomic model construction for supporting clinical translation [9].

Previous studies have extensively studied the reproducibility and robustness of radiomic features affected by various factors [10,11]. These factors can be divided into three classes, namely image acquisition factors, radiomic- or texture-specific image processing and feature computation. Studies on PET image acquisition factors include patient status (e.g., motion, respiratory motion) [12,13,14], image acquisition modes (e.g., scanner difference, test-retest repeatability) [15], image reconstruction [16,17,18], tumor delineation [19,20]. Studies on radiomic- or texture-specific image processing include image interpolation or voxel harmonization [21], and discretization methods or levels [22,23]. Although these studies analyzed and offered recommendations for optimizing features robustness (e.g., removing unstable features, using feature-wise preprocessing, large tumor volume definition), their investigated factors to determine radiomic features are still subject to variability, and did not involve the fundamental computation of features [24].

The computation of radiomic features, even with the same feature names, may be implemented differently in radiomic studies [25]. It not only depends on the parameters setting (such as symmetry, averaging strategy and distance) of texture matrices (e.g., grey-level co-occurrence matrix, GLCM; neighborhood grey tone difference matrix, NGTDM; and grey level zone size matrix, GLZSM), but also depends strongly on the aggregation methods of matrices. The concept of aggregation describes the process that combines multiple matrices (e.g., extracted from each single slice) to merge as one matrix for comprehensive representation of 3D tumor volume. Different parameter settings and aggregation methods can lead to different texture feature distributions and clinical characterization [26]. Hatt et al., first compared the strategies of averaging features from 13 independent matrices from each of 13 directions, versus directly calculating from one matrix considering 13 directions simultaneously [27]. Our previous study comprehensively investigated the impact of parameters (including symmetry, averaging strategy, and neighborhood extent or window size) of 3D textural matrix design on robustness and diagnostic performance of the resulting features [28]. Despite many significant suggestions, it only focused on parameter settings as generation of texture matrices.

In fact, textural features can be extracted from the largest cross-sectional (axial) slice of the tumour boundary (2D textures) or extracted from the entire tumour volume (3D textures). As reviewed by Reiazi et al. [29], although most clinical studies (about 62.2%) used 3D radiomics features, there are still 17.8% of studies that applied 2D features for radiomics analysis, and 20% of studies did not report any details on the dimension of textures. In [30], the author reported the better quantification of tumor heterogeneity in predicting clinical outcomes of 3D radiomic features compared to 2D features. A recent study [31] not only extracted radiomics features from 2D slice and 3D volume, but also averaged the features from each slice separately within the 3D tumor volume, namely 2.5D aggregation strategy. Their results showed that the overall performance of features under 2.5D aggregation strategy did not exceed based single 2D slice. Thus, there remains a long-time divergence about whether to use 2D or 3D annotations in specific radiomics-based research.

The Image Biomarker Standardization Initiative (IBSI) sought to standardize the radiomic analysis for improved clinical translation and provided a guideline to standardize the definition and computation of 174 radiomic features, including 95 texture features of six feature families under different aggregation methods [24]. It gives the standardized parameter settings and aggregation methods of matrices. For gray level co-occurrence matrix (GLCM) and Gray level run length matrix (GLRLM), the IBSI provides six aggregation methods for different dimensional features (2D, 2.5D and 3D). For gray level size zone matrix (GLSZM), gray level distance-zone matrix (GLDZM), neighborhood gray tone difference matrix (NGTDM) and neighborhood gray level dependence matrix (NGLDM), it provides three aggregation methods (2D, 2.5D and 3D). However, the impact of different aggregation methods on the computation of texture features has not been thoroughly evaluated.

The objective of the present work was therefore to evaluate the impact of aggregation method on the computation of texture features in [^18^F]FDG PET imaging of nasopharyngeal carcinoma patients. The consensus of two expert physicians for delineation was used as reference. This study investigates, in a comprehensive manner, the impact of variations in a range of aggregation methods used in the generation of 95 texture features. Robustness was assessed by the pair-wise intra-class correlation coefficient (ICC). The effect of different discretization and partial volume correction on the aggregation methods was further evaluated.

## 2. Materials and Methods

### 2.1. Patients and PET/CT Imaging

128 NPC patients (103 men and 25 women; mean age, 47.7 ± 13.2 years) with pathology confirmation (scan dates from January 2012 to August 2016) were enrolled in this study. The characteristics of 128 patients were summarized in Table 1. Under the European Association of Nuclear Medicine (EANM) procedure guidelines [1], patients underwent a pre-treatment whole-body [^18^F]FDG PET/CT scanning on a Biograph-128 mCT scanner (Siemens Healthineers, Shenzhen, Guangzhou). Furthermore, all patients underwent an additional local tumor imaging. To minimize the motion blur of the PET/CT system, patients were encouraged to lie still during the examination. Patients fasted for 6 h before radiotracer injection, and 306–468 MBq (8.27–12.65 mCi) of [^18^F]FDG (~150 μCi/kg of body weight) was administered intravenously for about 60 min imaging (mean: 58 ± 5 min, range [52–66 min]). CT scans (80 mA, 120 KVp) were used for attenuation correction. PET images were reconstructed using standard ordered-subset expectation maximization (OSEM) with three iterations and 21 subsets. PET images were reconstructed with matrix size of 200 × 200 and voxel size of 4.07 × 4.07 × 5 mm^3^. Then the PET image was cubic interpolated to the same dimension as CT matrix size of 512 × 512 and voxel sizes of 0.98 × 0.98 × 3 mm^3^ for image registration and fusion. The interpolated images were used for tumor delineation and radiomics features extraction. The body weight SUV images were calculated from original PET images with the formula as follows (Formula (1)):

(1) $$SUV(g/mL)\;=\frac{tissue\;activity(Bq/mL)}{injected\;dose(Bq)/body\;weight(g)}$$

where the tissue activity was decay-corrected to account for the time elapsed between injection and acquisition.

### 2.2. Tumor Delination, Partial Volume Correction and Intensity Discretization

For each patient, the PET/CT fusion images were displayed in ITK-SNAP software v.3.8 (http://www.itksnap.org (accessed on 29 January 2023)) with the horizontal-, coronal- and sagittal-views for visualization. Then two radiologists with 3 and 10 years of experience independently performed the delineation of primary tumors. High consistency with a median Dice Similarity Coefficient (DSC) of 0.87 was derived from the two 3D primary tumors. Leijenaar et al., first validated that features were more robust to inter-observer variability compared to test-retest variability [32]. Our previous study also validated that features were more robust with respect to different segmentation methods than discretization [23]. Therefore, the intersections of the two manual delineations by the two radiologists were used for radiomics analysis.

In order to evaluate the effect of partial volume correction on the robustness of aggregation methods, the Van Cittert deconvolution algorithm was applied on the original SUV images [33]. The Van Cittert iteration to estimate SUV is given as follows (Formula (2)):

(2) $$SUV^{\left(i\right)}=SUV^{\left(i-1\right)}+\alpha \left(SUV^{\left(0\right)}-\sigma ⊗SUV^{\left(i-1\right)}\right),SUV^{\left(i\right)}\geq 0$$

where $SUV^{\left(i\right)}$ is the ith estimation of SUV, and $SUV^{\left(0\right)}$ is the original SUV image, α is a parameter of order 1 that affects the convergence rate, σ is a normalized point spread function, ⊗ is the three-dimensional convolution operator. The only constraint on $SUV^{\left(i\right)}$ is that each voxel value must be positive. The VC deconvolution was applied on the SUV image with fixed 10 iterations and varied σ (from 1 to 5).

The SUVs of each VOI were then resampled into D bins [22], as follows (Formula (3)):

(3) $$SUV_{D}(x)=\left\{\begin{array}{ll} 1 & SUV(x)=SUV_{\min}\\ \left\lceil D\times \frac{SUV(x)\;-SUV_{\min}}{SUV_{\max}-SUV_{\min}}\right\rceil & otherwise \end{array}\right.$$

where SUV(x) is the SUV of voxel x, $SUV_{D}(x)$ is the resampled value of voxel x. The SUV resolution equals $\left(SUV_{\max}-SUV_{\min}\right)/D$. Discretization was performed with a fixed bin number (e.g., D= 8, 16, 32, 64 and 128), namely FBN. The discretization step is necessary to generate texture matrices, and bin number determinates the intensity resolution and the corresponding computation complexity of texture matrices.

### 2.3. Matrix Construction with Different Aggregation Methods

**Figure 1:**
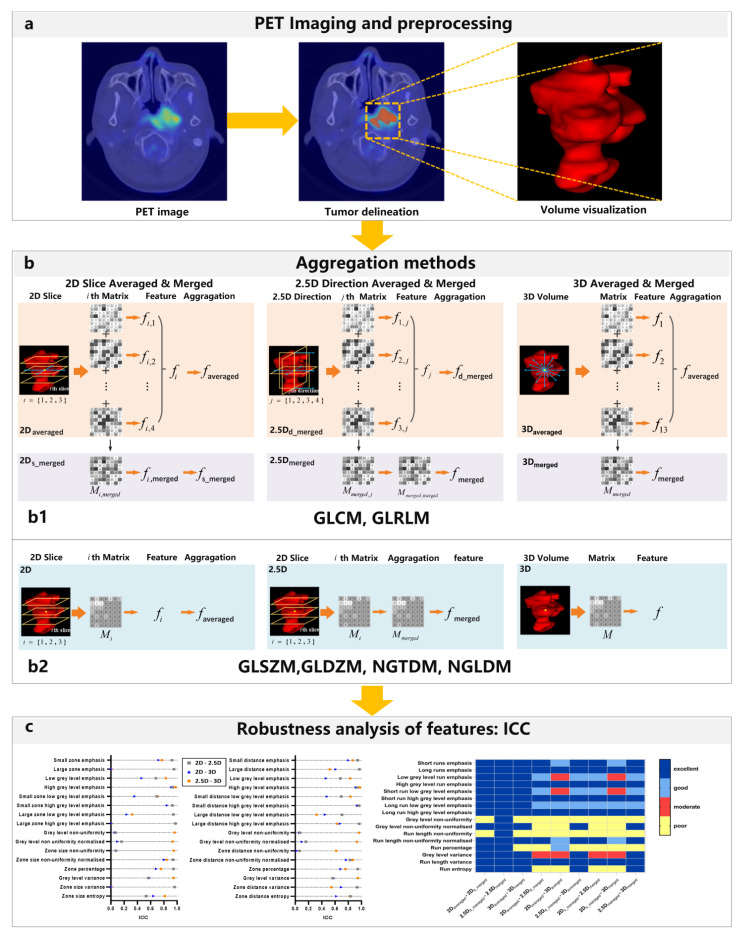
The flowchart of the present study includes PET imaging and processing, aggregation methods, and the robustness analysis of features. (**a**) Illustration of PET/CT imaging of nasopharyngeal carcinoma patients (NPC) and the corresponding segmentation. (**b**) Aggregation methods for textural matrices, (**b1**) six aggregation methods of GLCM and GLRLM, which is composed by three classes (namely 2D slice-, 2.5D direction-, and 3D-averaged & merged), and each class includes two aggregation methods (namely features averaged and matrices merged), (**b2**) three aggregation methods of GLSZM, GLDZM, NGTDM, NGLDM. (**c**) Robustness analysis of features, including pair-wise ICC and ICC heat map.

The flowchart of the study design is shown in Figure 1. A schematic example of feature aggregation is shown in Figure 1b. According to IBSI guideline [24], the gray level co-occurrence matrix (GLCM) is generated by considering the occurrence of two pixels with intensity i and j separated by fixed distance D = 1 in direction θ. To improve rotational invariance, GLCM features are computed by aggregating information from the different underlying directional matrices. Six specific aggregation methods of GLCM and GLRLM are defined as follows:

- Features are computed from each 2D directional matrix and then averaged over 2D directions and slices, namely 2D_averaged_;
- Features are computed from a single matrix after merging 2D directional matrices per slice, and then averaged over slices, namely 2D_s-meraged_;
- Features are computed from a single matrix after merging 2D directional matrices per direction, and then averaged over directions, namely 2D_d-meraged_;
- The feature is computed from a single matrix after merging all 2D directional matrices, namely 2D_meraged_;
- Features are computed from each 3D directional matrix and averaged over the 3D directions, namely 3D_averaged_;
- The feature is computed from a single matrix after merging all 3D directional matrices, namely 3D_meraged_.

Three aggregation methods in calculating GLSZM, GLDZM, NGTDM, and NGLDM are defined as follows:

- Features are computed from 2D matrices and averaged over slices, namely 2D;
- The feature is computed from a single matrix after merging all 2D matrices, namely 2.5D;
- The feature is computed from a 3D matrix, namely 3D.

### 2.4. Feature Extraction

In short, after the construction of matrices with different aggregation methods as shown in Table 2, 25, 16, 16, 16, 5 and 17 features were extracted from GLCM, GLRLM, GLSZM GLDZM NGTDM and NGLDM under each aggregation method, respectively.

**Table 1:** Clinical characteristics of the NPC patients.

Characteristic	All Patients
Patient No.	128
Age (year), mean ± SD	47.7 ± 13.2
Sex, no.(%)	
Male	103 (80.5%)
Female	25 (19.5%)
AJCC stage, no.(%)	
I	4 (3.1%)
II	11 (8.6%)
III	49 (38.3%)
IV	64 (50%)
MATV	50.9 ± 86.4
SUVmax	15.4 ± 7.77
SUVmean	7.95 ± 3.75
CTmean	50.4 ± 55.1

### 2.5. Robustness Evaluation by Intra-Class Coefficient (ICC)

In order to analyze the robustness of feature values calculated with different aggregation methods, the intra-class coefficient (ICC) [34] was adopted (Formula (4)):

(4) $$\mathrm{ICC}=\frac{\mathrm{BMS}-\mathrm{WMS}}{\mathrm{BMS}+WMS}$$

where *BMS* and *WMS* were the between-subjects and within-subjects mean squares, obtained via Kruskal-Wallis one-way ANOVA. ICC ranges from 0 to 1. The higher the ICC, the more robust the feature and an ICC of one indicates perfect robustness (i.e., identical feature values). The textural features were categorized as having poor (ICC < 0.5), moderate (0.5 ≤ ICC < 0.75), good (0.75 ≤ ICC < 0.9), or excellent (ICC ≥ 0.9) robustness.

We first used pair-wise ICC to investigate the impact of aggregation methods as used for the generation of texture features on their robustness. The features were extracted with fixed discretization level (FBN = 128) from original PET images. The overall ICC calculated by considering all the aggregation methods for each feature was used to validate that if our results will be affected by different discretization levels (FBN = 8, 16, 32, 64, 128) and partial volume correction (varying normalized point spread function).

Furthermore, under different discretization levels and partial volume corrections, the relationships between features with excellent robustness and MATV were studied to assess the potential complementarity of texture features to MATV. Since such relationships are nonlinear and these parameters frequently are not normally distributed, Spearman rank correlation (rs) was exploited in this study.

## 3. Results

### 3.1. Robustness of GLCM Features Affected by Aggregation Methods

**Figure 2:**
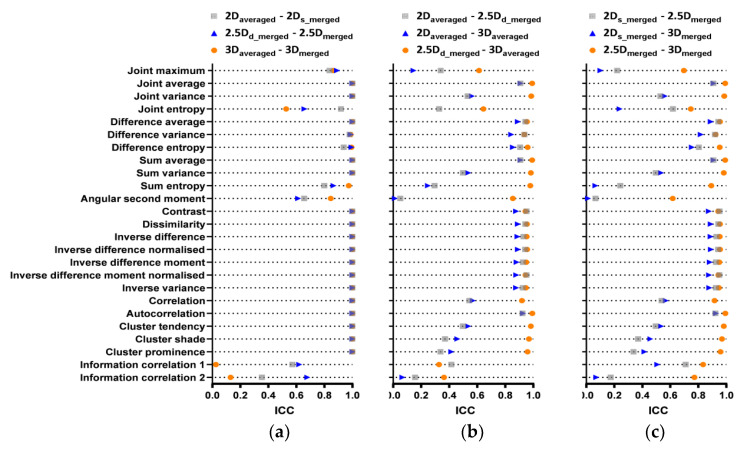
Pairwise ICC for each GLCM feature extracted from: (**a**) 2D_averaged_-2D_s_merged_, 2.5D_d_merged_-2.5D_merged_, 3D_averaged_-3D_merged_, (**b**) 2D_averaged_-2.5D_d_merged,_ 2D_averaged_-2.5D_merged_, 2.5D_d_merged_-3D_averaged_, (**c**) 2D_s_merged_-2.5D_merged_, 2D_s_merged_-3D_merged_, and 2.5D_merged_-3D_merged_.

Ideally, there are six aggregation methods for GLCM, and corresponding $C_{6}^{2}=15$ pairs of aggregation methods. Considering that some pairs of aggregation methods (simultaneously with different feature matrix dimension and averaged/merged strategies) are meaningless, we only evaluated nine pairs of aggregation methods with the same feature matrix dimensions or same averaged/merged strategies. Figure 2 illustrates pair-wise ICC values of GLCM texture features as extracted from FBN = 128 for nine pairs of aggregation methods. Twenty-one features showed ICC values of larger than 0.75 (19/21 features depicted ICC values of nearly 1) among the three combination strategies of six aggregation methods (2D_averaged_-2D_s_merged_, 2.5D_d_merged_-2.5D_merged_, and 3D_averaged_-3D_merged_), while four features showed ICC values lower than 0.75 as shown in Figure 2a. Thus, the effect of aggregation methods on the same dimension (2D, 2.5D, and 3D) feature was negligible except for four features (Joint entropy, Angular second moment, Information correlation1 and correlation2). Thirteen features showed ICC values of larger than 0.75 among the three combination strategies of six aggregation methods (2D_averaged_-2.5D_d_merged_, 2D_averaged_-3D_averaged_, and 2.5D_d_merged_-3D_averaged_), while twelve features showed parts or all ICC values lower than 0.75 as shown in Figure 2b. Thus, the effect of feature matrix dimension on similar aggregation methods (d_merged can be considered as an averaged strategy) was not negligible. The same conclusion could be derived from Figure 2c.

**Figure 3:**
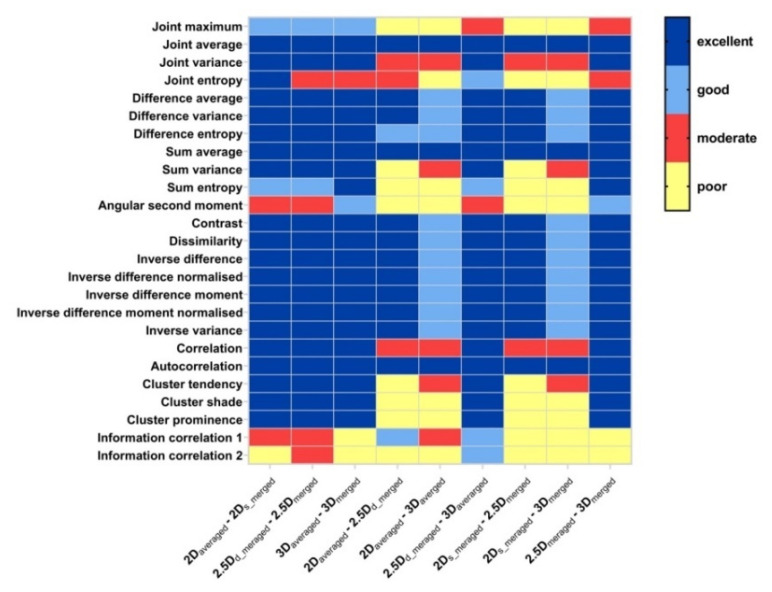
ICC heat map of GLCM features; volumes: pair-wise aggregation methods corresponding to Figure 2; rows; GLCM features. Blue = ICC > 0.9 (excellent), light blue = 0.75 ≤ ICC < 0.9 (good), red = 0.5 ≤ ICC < 0.75 (moderate), yellow = ICC < 0.5 (poor).

Figure 3 illustrates ICC heat map of GLCM features. For features extracted with different aggregation methods for the same dimension (Figure 3 left), we can see that the effect of aggregation methods on feature values was negligible for most features (with excellent robustness) except for four features (joint entropy, angular second moment, information correlation1 and correlation2). For features extracted with the same aggregation methods (d_merged can be considered as an averaged strategy) for the different dimension (Figure 3 middle and right), it is worth noting that 3/25 features (joint average, sum average, autocorrelation) showed excellent robustness, and 10/25 features (difference average, difference variance, difference entropy, contrast, dissimilarity, inverse difference, inverse difference moment, inverse difference moment normalized, inverse variance) showed good or excellent robustness.

### 3.2. Robustness of GLRLM Features Affected by Aggregation Methods

**Figure 4:**
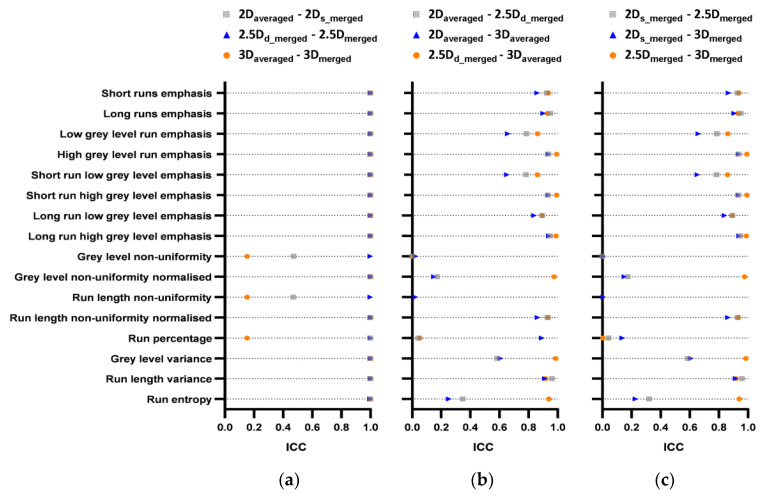
Pairwise ICC for each GLRLM feature extracted from: (**a**) 2D_averaged_-2D_s_merged_, 2.5D_d_merged_-2.5D_merged_, and 3D_averaged_-3D_merged_, (**b**) 2D_averaged_-2.5D_d_merged_ 2D_averaged_-2.5D_merged_, and 2.5D_d_merged_-3D_averaged_, and (**c**) 2D_s_merged_-2.5D_merged_, 2D_s_merged_-3D_merged_, and 2.5D_merged_-3D_merged_.

Figure 4 illustrates pair-wise ICC values of GLRLM texture features as extracted from FBN = 128 for nine pairs of aggregation methods. Comparing the aggregation methods in same dimension (2D_averaged_ vs. 2D_s_merged_, 2.5D_d_merged_ vs. 2.5D_merged_, and 3D_averaged_ vs. 3D_merged_), 13/16 features depicted high robustness with the ICC close to one as shown in Figure 4a. In particular, all 16 features have the nearly the same values between the aggregation methods of 2.5D_d_merged_ and 2.5D_merged_, which indicated a negligible effect of aggregation methods. Seven features showed an ICC larger than 0.75 when considering the three pairs of 2D_averaged_ vs. 2.5D_d_merged_, 2D_averaged_ vs. 3D_averaged_, 2.5D_d_merged_ vs. 3D_averaged_ (Figure 4b, while 9/16 features showed moderate robustness with an ICC lower than 0.75. The results suggested that the different dimensions of aggregation methods in calculating textural matrices led to the relatively large difference of features values. Furthermore, we found that the robustness of GLRLM features for the same dimension affected by different aggregation methods is closed, as shown in Figure 4b,c.

**Figure 5:**
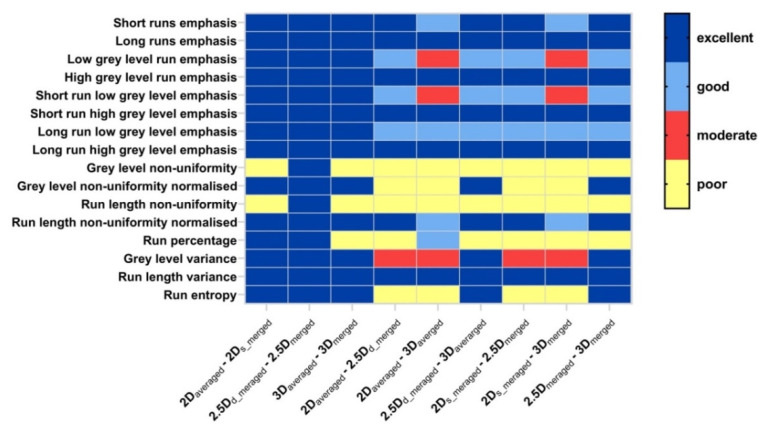
ICC heat map of GLRLM features; volumes: pair-wise aggregation methods corresponding to Figure 2; rows; GLCM features. Blue = ICC > 0.9 (excellent), light blue = 0.75 ≤ ICC < 0.9 (good), red = 0.5 ≤ ICC < 0.75 (moderate), yellow = ICC < 0.5 (poor).

Figure 5 illustrates the ICC heat map of GLRLM features. For features extracted with different aggregation methods for the same dimension (Figure 5 left), we can see that the effect of aggregation methods on the same dimension (2D, 2.5D, and 3D) features was negligible. There are only 3/16 features showed poor or moderate robustness. For features extracted with same aggregation methods (d_merged can be considered as an averaged strategy) for different dimensions (Figure 5 middle and right), we can also note that there are 5/16 features (long runs emphasis, high grey level run emphasis, short run high grey level emphasis, long run high grey level emphasis, run length variance) showed excellent robustness, and 3/16 features (short runs emphasis, long run low grey level emphasis, run length non-uniformity normalized) showed good or excellent robustness.

### 3.3. Robustness of GLSZM, GLDZM, NGLDM, NGTDM Features Affected by Aggregation Methods

**Figure 6:**
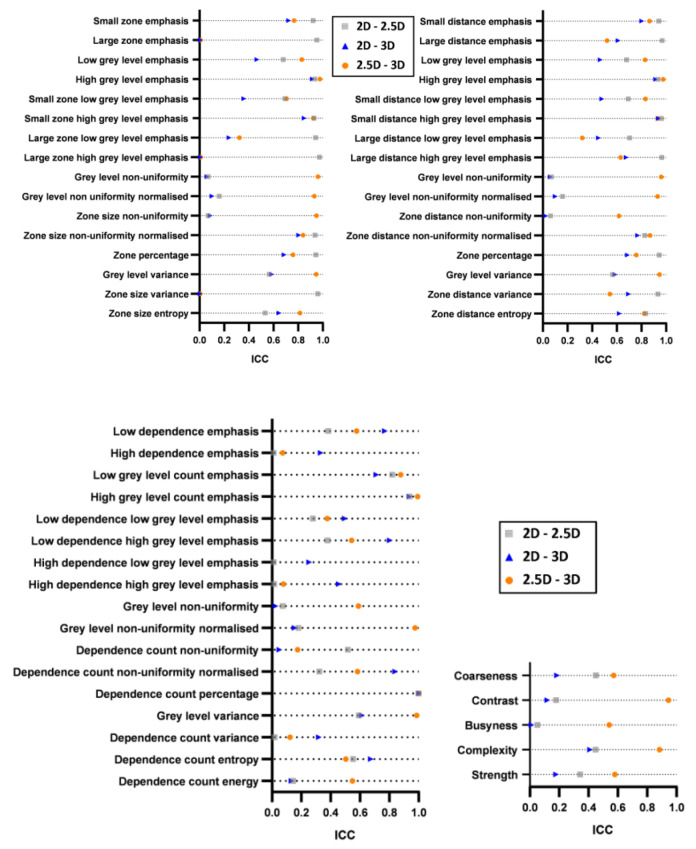
Pairwise ICC for each of GLSZM (**top-left**), GLDZM (**top-right**), NGLDM (**bottom-left**) and NGTDM (**bottom-right**) features extracted from three aggregation strategies (2D, 2.5D, 3D), denoted as 2D-2.5D, 2D-3D, and 2.5D-3D.

Figure 6 shows pairwise ICC for GLSZM (top-left), GLDZM (top-right), NGLDM (bottom-left) and NGTDM (bottom-right) features extracted from three aggregation strategies (2D, 2.5D, 3D), denoted as 2D-2.5D, 2D-3D, and 2.5D-3D. Nine of the 16 features of GLSZM (Figure 6 top-left) showed ICC values of larger than 0.75 among the combination of two aggregation methods (2D-2.5D). Another 9/16 features of GLSZM showed ICC values of larger than 0.75 among the combination of two aggregation methods (2.5D-3D). Only 3/16 features of GLSZM showed ICC values of larger than 0.75 among the combination of two aggregation methods (2D-3D). This indicates that the effect of the feature matrix dimensions (2D-3D) is larger than 2D-2.5D and 2.5D-3D. Nine out of 16 features of GLDZM (Figure 6 top-right) showed ICC values of larger than 0.75 among the combination of two aggregation methods (2D-2.5D). Another 10/16 features of GLDZM (Figure 6 top-right) showed ICC values of larger than 0.75 among the combination of two aggregation methods (2.5D-3D). Only 3/16 features of GLDZM showed ICC values of larger than 0.75 among the combination of two aggregation methods (2D-3D). Similar conclusions are derived that the effect of the feature matrix dimensions (2D-3D) is larger than 2.5D-3D and 2D-2.5D. Figure 6 shows the pairwise ICC for each NGTDM (bottom-right) and NGLDM (bottom-left) features extracted from three aggregation strategies (2D, 2.5D, 3D), denoted as 2D-2.5D, 2D-3D, and 2.5D-3D. There are only two features and no features showed ICC values of larger than 0.9 for NGLDM (bottom-left) and NGTDM (bottom-right) features.

**Figure 7:**
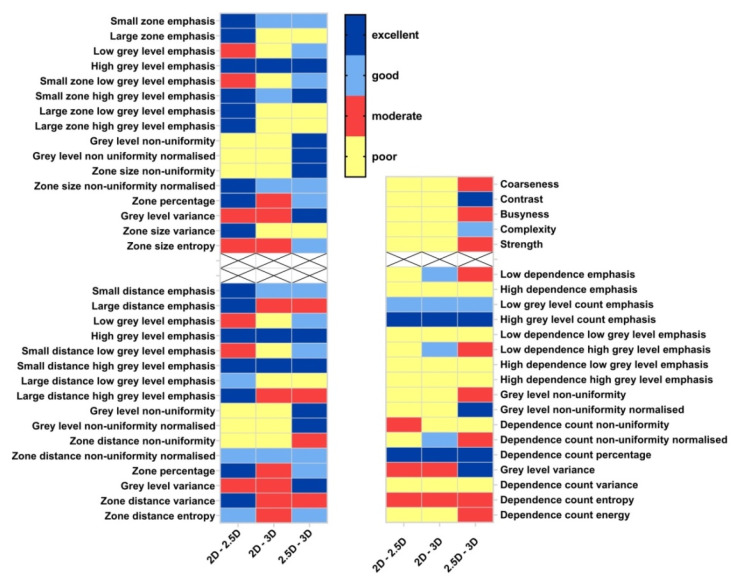
ICC heat map of GLSZM (**top-left**), GLDZM (**bottom-left**), NGTDM (**top-right**) and NGLDM (**bottom-right**) features; volumes: pair-wise aggregation methods corresponding to Figure 2; rows; GLCM features. Blue = ICC > 0.9 (excellent), light blue = 0.75 ≤ ICC < 0.9 (good), red = 0.5 ≤ ICC < 0.75 (moderate), yellow = ICC < 0.5 (poor).

Figure 7 illustrates the ICC heat map of GLSZM (top-left), GLDZM (bottom-left), NGTDM (top-right) and NGLDM (bottom-right) features. Based on the ICC value, textural features can be divided into sub-groups with excellent, good, moderate and poor robustness, respectively. There are only five features (high grey level emphasis from GLSZM, high grey level emphasis and small distance high grey level emphasis from GLDZM, high grey level count emphasis and dependence count percentage from NGLDM) that showed excellent robustness. Six features showed excellent or good robustness, including three GLCM features (small zone emphasis, small zone high grey level emphasis, zone size non-uniformity normalized), two GLDZM features (small distance emphasis, Zone distance non-uniformity normalized) and one NGLDM feature (low grey level count emphasis).

### 3.4. Effect of Discretization and Partial Volume Correction on Feature’s Robustness

**Figure 8:**
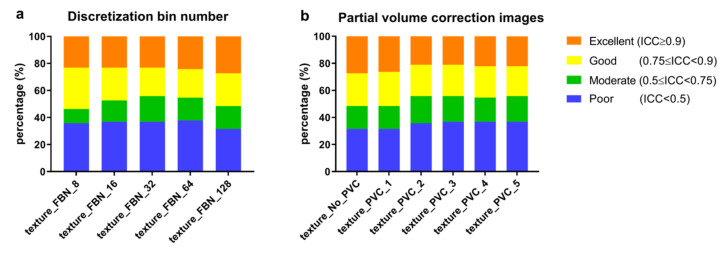
Agreement of texture features between different aggregations methods from: (**a**) original SUV image discretized with FBN from 8 to 128, and (**b**) original SUV and PVC images (with FWHM from 1 to 5) discretized with FBN = 128. The relative distribution of the overall ICC value of texture features per ICC category are shown (ICC > 0.9: excellent, 0.75 ≤ ICC < 0.9: good, 0.5 ≤ ICC < 0.75: moderate, ICC < 0.5: poor).

To evaluate the effect of discretization and partial volume effect on the percent of ICC categories of all texture features, instead overall ICC was computed of the pair-wise ICC to simplify the computation. From Figure 8a, different discretization levels lead to consistent robustness of textural features affected by aggregation methods. Similarly, different FWHM parameters of PVC also hardly change the robustness of features. The results showed that different discretization levels and PVC algorithms have a negligible effect on the percent of ICC categories of all texture features.

**Figure 9:**
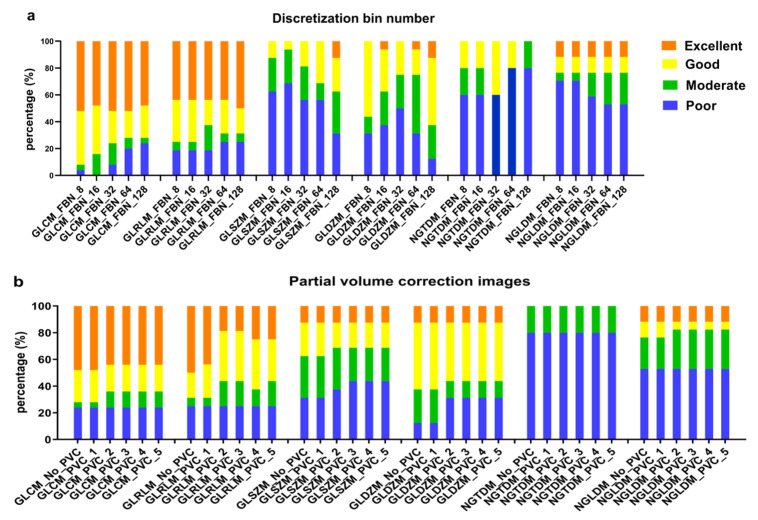
Agreement of each features families between different aggregations methods from: (**a**) original SUV image discretized with FBN from 8 to 128, and (**b**) original SUV and PVC images (with FWHM from 1 to 5) discretized with FBN = 128. The relative distributions of the overall ICC value of texture features per ICC category are shown (ICC > 0.9: excellent, 0.75 ≤ ICC < 0.9: good, 0.5 ≤ ICC < 0.75: moderate, ICC < 0.5: poor).

The results of overall ICC for each feature family are shown in Figure 9. For all feature families, discretization levels exhibit a larger effect than partial volume correction with larger changes of relative distribution of the overall ICC per ICC category. For GLCM and GLRLM based features, most features had an excellent or good agreement between different aggregation methods with increasing FBN from 8 to 128. For GLSZM, GLDZM, NGTDM and NGLDM, most features had a moderate or poor agreement between different aggregation methods with increasing FBN from 8 to 128. For all feature families, most features had a fixed agreement between different aggregations with PVC images (FWHM from 1 to 5), and only a few features (GLRLM) had decreased excellent agreement with increasing FWHM of PVC.

### 3.5. Effect of Discretization and Partial Volume Correction on Correlation between Features with Excellent Robustness and MATV

**Figure 10:**
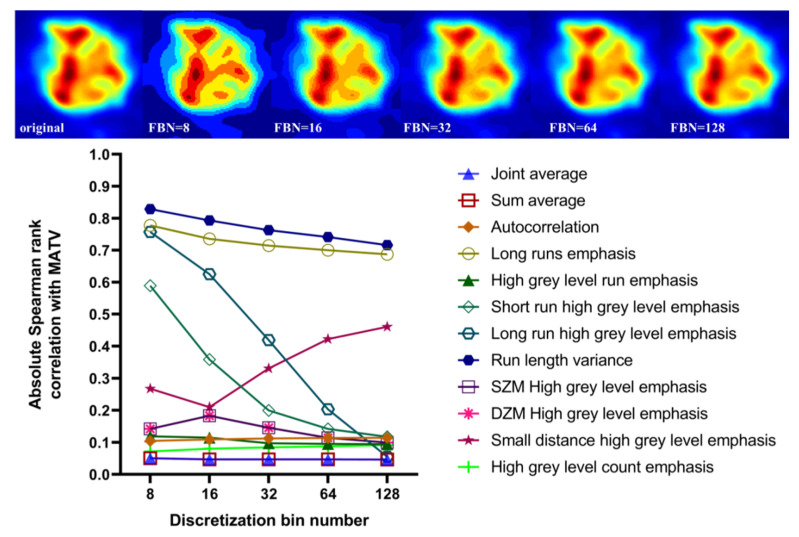
Correlation between features with excellent robustness and MATV from: original SUV image discretized with FBN from 8 to 128.

To avoid confusion, an absolute Spearman rank correlation is reported and correlation direction results can be found in Figure 10. First, significant details on the gray-level distribution will be preserved when using a discretization of FBN more than 64. We can see that the discretization has an important impact on the correlation between features (run length variance, long run emphasis, long run high grey level emphasis, short run high grey level emphasis) and MATV, decreasing with the FBN increasing. The correlation of seven features (joint average, sum average, autocorrelation, high grey level run emphasis, SZM high grey level emphasis, DZM high grey level emphasis, high grey level count emphasis) was insensitive to the quantization values. Only the correlation between feature (small distance high grey level emphasis) and MATV increased with the FBN increasing. The correlation between features and MATV ranged from 0.04 to 0.82, suggesting that a substantial amount of complementary information with respect to MATV may be found in some of these texture features.

**Figure 11:**
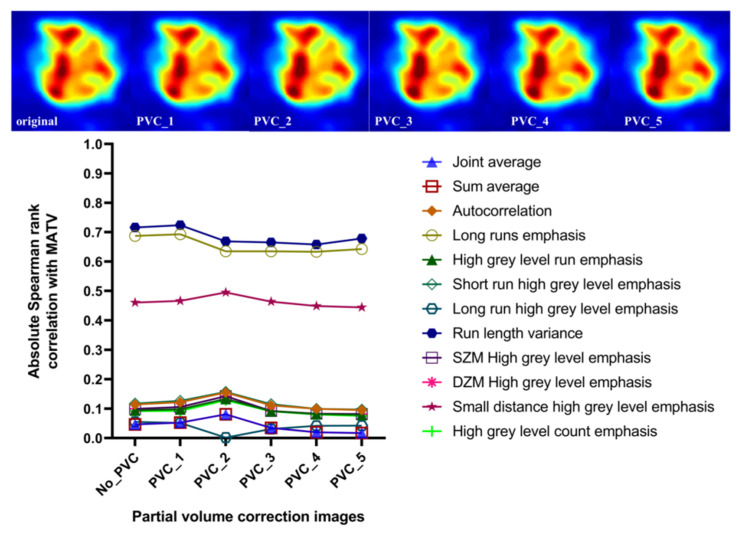
Correlation between features with excellent robustness and MATV from: original SUV and PVC images (with FWHM from 1 to 5) discretized with FBN = 128.

As shown in Figure 11, the high uptake region will be enhanced with increasing FWHM of PVC. However, the PVC has a negligible effect on the correlation between all features and MATV, and all the rs values have a very small variation with increasing FWHM of PVC.

## 4. Discussion

The present study focused on the internal factor, namely the aggregation methods, for the construction of texture feature matrices. We assessed the impact of aggregation methods as used in the generation of texture features on their robustness in nasopharyngeal PET/CT. Furthermore, the effect of different discretization and partial volume correction on the distribution of ICC was further evaluated.

### 4.1. The Research Value of This Study

Different software tools can produce noticeably different values for the same image biomarker. It would be difficult to compare, for consistency and standardization, the radiomic features extracted by different software implementations and details. The IBSI has made great progress in minimizing such difference, and variance due to software will no longer be important [24]. As reviewed in [29], one ambiguity in radiomics analysis was the aggregation methods of feature computation. Though IBSI gives definite aggregation methods for each feature family, one question has not been answered is the impact of the aggregating strategy for texture features on their robustness performance. The present study aims to investigate the impact of aggregation methods as used for the generation of texture features on their robustness of nasopharyngeal carcinoma (NPC) based on ^18^F-FDG PET/CT images. The objective was two-fold. First, various features have been selected for specific task in a previous study [35,36,37]. However, the same features could have different values in different studies, and present results can give guidelines for choosing the robust features for different aggregation methods. Second, the features with excellent and good robustness could be used for stable model construction, which is one of the possible research topics of radiomics [38].

### 4.2. The Effect of Different Aggregation Methods and Feature Dimension on the Feature’s Robustness

For GLCM and GLRLM, the effect of different aggregation methods (averaged and merged) on the same dimensional features (2D, 2.5D or 3D) was negligible despite the fact that six features (joint entropy, angular second moment, information correlation 1, information correlation 2, grey level non-uniformity, run length non-uniformity) were not robust. This conclusion almost agrees with our previous study [28], that the effect of combination strategies on the 3D feature is negligible or the 3D feature was robust to different combination strategies with higher ICC. This suggests that most of the same dimensional GLCM and GLRLM based features can be exploited by ignoring the aggregation methods except for the after-mentioned six features.

The effect of the same aggregation method (averaged or merged) on different dimensional features (2D-2.5D, 2D-3D, 2.5D-3D) showed similar robustness performance. Obviously, different dimensional features with same aggregation methods showed worse robustness compared with the same dimensional features with different aggregation methods. This conclusion was first derived from GLCM and GLRLM based features, and then validated from the other feature families. In the present work, the clinical usefulness of the feature has been evaluated, and the reason is that the choice of clinically useful features should be based on the specific task (i.e., diagnosis, prognosis, etc.), as in our previous study [28]. The aim of this study is to provide a guideline/reference for researchers to choose a stable/robust feature when varying the aggregation methods.

### 4.3. Statistical Metric and Cutoff Values

Feature robustness was assessed by the intra-class correlation coefficient (ICC). The ICC metric is appropriate where one expects a strong correlation with a given class but a weak correlation between classes. However, the determination of a robust feature is depended on the specific cutoff values of ICC, which segregate stable from unstable features. Different studies give different threshold values to define a feature as highly reproducible, such as ICC ≥ 0.9 [18,39] and ICC ≥ 0.8 [23,28] in our previous study. This leads to differences in the individual features and there was no universal consensus. In the present study, multiple thresholds were simultaneously given and features were categorized as having poor (ICC < 0.5), moderate (0.5 ≤ ICC < 0.75), good (0.75 ≤ ICC < 0.9), or excellent (ICC ≥ 0.9) robustness, as adopted in [40]. This method can provide the distribution of feature robustness from poor to excellent other than simply distinguish the feature robustness based on single thresholds.

### 4.4. The Effect of Discretization and Partial Volume Corrections on the Percent of ICC Categories of All Texture Features

Discretization reduces the infinite possible number of intensity values to a finite set and effectively reduces image noise [41]. Commonly, there are two discretization methods, namely fixed bin number (FBN) and fixed bin size (FBS) [42]. The FBN methods divide the image SUV range into fixed bin number, this results in discretized images with varying bin sizes, depending on the SUV range of each ROI. The FBS methods divide the image SUV range into uniform bin size and maintain a constant intensity resolution across all tumor images [22]. In the present study, FBN = 128 was adopted in the experiment design as in [43], which is enough to describe the grey level of a tumor and has acceptable computation complexity. Of course, we also set the FBN from 8 to 128 to evaluate the effect of discretization on the percent of ICC categories of all texture features. The results showed that different discretization levels have a negligible effect on the percent of ICC categories of all texture features. For each feature family, there is a small disagreement of ICC categories between different discretization levels; the main reason is that the discretization of FBN ≤ 32 will not be enough to describe the grey level distribution, which will finally affect the feature computation [44].

Accurate quantification of metabolic volumes of PET is hampered by partial volume effects, leading to underestimation of the standardized uptake value (SUV) [45]. Various partial volume correction methods have been advocated for [46], mainly including enhancing spatial resolution during reconstruction and post reconstruction image restoration. In the present study, the classic Van Cittert (VC) deconvolution technique was adopted as a partial volume correction [33], which is relatively effective and simple to apply with only one convolution kernel parameter compared to reconstruction-based methods. We also set the kernel parameters from 1 to 5, which means simultaneously increasing the SUV uptake and noise level. Thus, we can evaluate the effect of partial volume corrections on the percent of ICC categories of all texture features. It is worth noting that the Lucy–Richardson method is as easy to implement as the Van Cittert method (the only difference is in the iteration step which is multiplicative in the Lucy–Richardson method and additive in the Van Cittert method) [47]. In our previous study, we also validate that there is no significant difference between these two methods [48]. Furthermore, there are also methods incorporating regularization or denoising term into LR or VC correction to control the noise, such as wavelet-based denoising [49], HYPR denoising [50], MR-guided regularization [51,52] and parallel level set regularization [53]. However, these methods will increase the additional regularization parameters and the optimization of these parameters is relatively difficult to achieve in clinical application.

### 4.5. Robustness and Discriminability of Features

In the present study, we aimed to investigate the impact of aggregation methods as used for the generation of texture features on their robustness of nasopharyngeal carcinoma (NPC) based on ^18^F-FDG PET/CT images. Thus, this study focused on the robustness or stability of features rather than the discriminability of features as in [15,16,17,18]. The robustness or stability of features was assessed by the intra-class correlation coefficient (ICC). The discriminability of the feature depends on the specific clinical task and machine learning algorithms, including feature selection methods and classifiers. In our previous study, we investigated 42 machine learning methods cross-combined from six feature selection methods and seven classifiers for radiomics-based differentiation of local recurrence versus inflammation from post-treatment NPC PET/CT images [54]. The results showed that three combinations from a Fisher score with random forests (RF), *k*-nearest neighborhood (kNN) and support vector machine (SVM) had a comparatively higher performance in distinguishing recurrence and inflammation. As a univariate selection method, the Fisher score can deal with feature relevance unable to detect feature redundancy. We also noted that a new feature selection algorithm, namely heat transfer search, which is proposed in [55] and applied in hybrid perovskites thin films morphology identification [56]. The combinations of four machine learning methods with a heat transfer search were evaluated and compared with a cuckoo search algorithm. The results showed that the features selected through the heat transfer search algorithm are effective in identifying thin film morphological images with machine learning models. Though the heat transfer search is applied in a different context, it has great potential application in radiomics. In the future, we will simultaneously evaluate the robustness and discriminability of features for a specific clinical task, and incorporate the novel heat transfer search into model construction.

### 4.6. Limitations

Our study had some limitations. First, we had 128 NPC patients in a single center and a relatively small data set to validate the robustness of the features. Ideally, multi-center and a large data set should be used for validation of the reliability of the results. In the present study, we used multiple preprocessing methods, including different discretization levels and partial volume corrections, to validate the consistency of our results. Secondly, we only considered different aggregation methods for all the features, and the parameter setting was a default as suggested in IBSI, which can make the conclusions more generalizable for most of researchers.

## 5. Conclusions

In the present study, we enrolled 128 NPC patients and extracted six feature families and a total of 95 texture features under different aggregation methods. Robustness was assessed by the pair-wise intra-class correlation coefficient (ICC). Only 12 features with excellent robustness with varying aggregation methods were selected from 95 textural features. Different dimensional features with the same aggregation methods showed worse robustness compared with the same dimensional features with different aggregation methods. Different discretization levels and PVC algorithms have a negligible effect on the percent of ICC categories of all texture features.

## Figures and Tables

**Table 2:** Feature families and corresponding feature number and aggregation methods.

Feature Family	Count	Aggregation Methods
GLCM	25	6
GLRLM	16	6
GLSZM	16	3
GLDZM	16	3
NGTDM	5	3
NGLDM	17	3

## Data Availability

The data presented in this study are available on request from the corresponding author.
